# Accidental Carbon Monoxide Poisoning Leading to Devastating Cardiovascular Consequences

**DOI:** 10.1016/j.jaccas.2025.103320

**Published:** 2025-03-12

**Authors:** Debanshu Roy, Daniel J. McGowan, Robert Chilton, Pankaj Kulshrestha

**Affiliations:** a Audie Murphy VA, San Antonio, Texas, USA; b Good Samaritan Hospital, Kearney, Nebraska, USA; c Audie L. Murphy Memorial Veterans Hospital, San Antonio, Texas, USA; d Good Samaritan Hospital, Kearney, Nebraska, USA

**Keywords:** acute limb ischemia, chemically induced disorders, left ventricular thrombus, myocardial infarction, pulmonary embolism, thrombosis

## Abstract

We report a case of a young male patient presenting with accidental carbon monoxide poisoning leading to pulmonary embolism, acute myocardial infarction, right and left ventricular thromboses, acute limb ischemia, and acute renal failure. We review clinical aspects of carbon monoxide poisoning with a specific focus on cardiovascular and systemic thrombotic complications.

## Graphical Abstract

**Figure:**
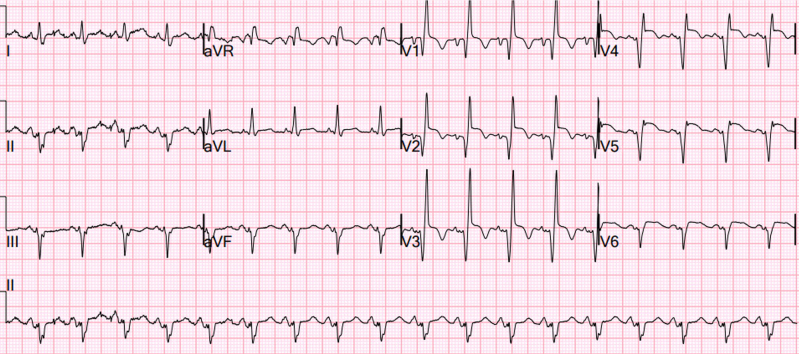

## History of Presentation

A 44-year-old male patient with a history of smoking presented to the emergency department with shortness of breath, confusion, right lower extremity coldness, pain, and paresthesia. He was found in a stuporous condition at his residence by his mother. Due to relatively higher incidences of carbon monoxide (CO) poisoning in rural Nebraska, especially during winter months, emergency first responders checked the environmental CO level, which was significantly elevated (300 parts per million [normal: 0-10 parts per million]).

**Take-Home Message**

• CO monoxide poisoning should be considered in the differential for “unprovoked” cases of venous/arterial/intracardiac thromboembolism.

The patient’s initial vital signs were as follows: temperature 100.6°F; blood pressure 116/70 mm Hg; pulse 90 beats/min; respiratory rate 20 breaths/min; and oxygen saturation level of 100% with 100% fraction of inspired oxygen. Physical examination revealed jugular venous distension, dynamic apical impulse, soft S1 and S2 sounds, and right lower extremity coldness with absent right dorsalis pedis pulse.

## Past Medical History

The patient had been diagnosed with “unprovoked” lower extremity deep vein thromboses and pulmonary embolism 2 months ago. He was treated with apixaban. However, the patient reported noncompliance with apixaban for the past 1 month.

## Differential Diagnosis

Simultaneous presentation of acute myocardial infarction *and* acute limb ischemia was indicative of cardio-embolism; atrial fibrillation, cardiac tumors/masses/vegetations, and catastrophic anti-phospholipid antibody syndrome were all plausible causes. The patient’s social history of CO leakage from a household water heater put CO poisoning in the differential diagnosis. Although acute myocardial infarction and acute limb ischemia could well be from cardio-embolism (ventricular thrombus), temporal proximity of venous (recent deep vein thrombosis and pulmonary embolism) and arterial thromboembolism (within 4-8 weeks) are indicative of an occult hypercoagulable state (CO exposure and noncompliance with apixaban). The patient’s normal platelet count and lack of evidence of hemolysis point against anti-phospholipid antibody syndrome. Anti-cardiolipin antibody/lupus anticoagulant tests were not checked.

## Investigations

**Figure 1:**
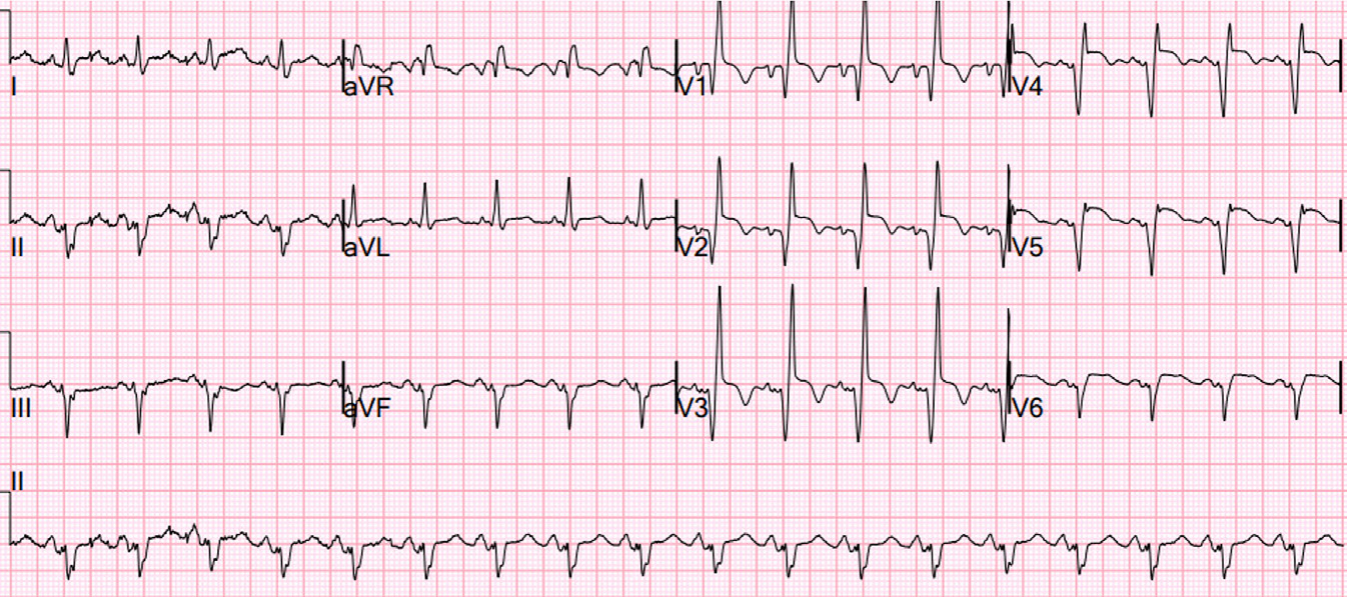
Initial ECG Showing Anterior STEMI ECG = electrocardiogram; STEMI = ST-segment elevation myocardial infarction.

The patient’s initial electrocardiogram was consistent with anterior ST-segment elevation myocardial infarction (Figure 1). Venous CO hemoglobin saturation was 29.9%, white blood cell counts 21.6 × 10^3^ /μL, hemoglobin 19.5 g/dL, and platelets 114,000/μL. High-sensitivity troponin level was 29,966 ng/L on day 1, which rose to 34,138 ng/L on day 2.

**Figure 2:**
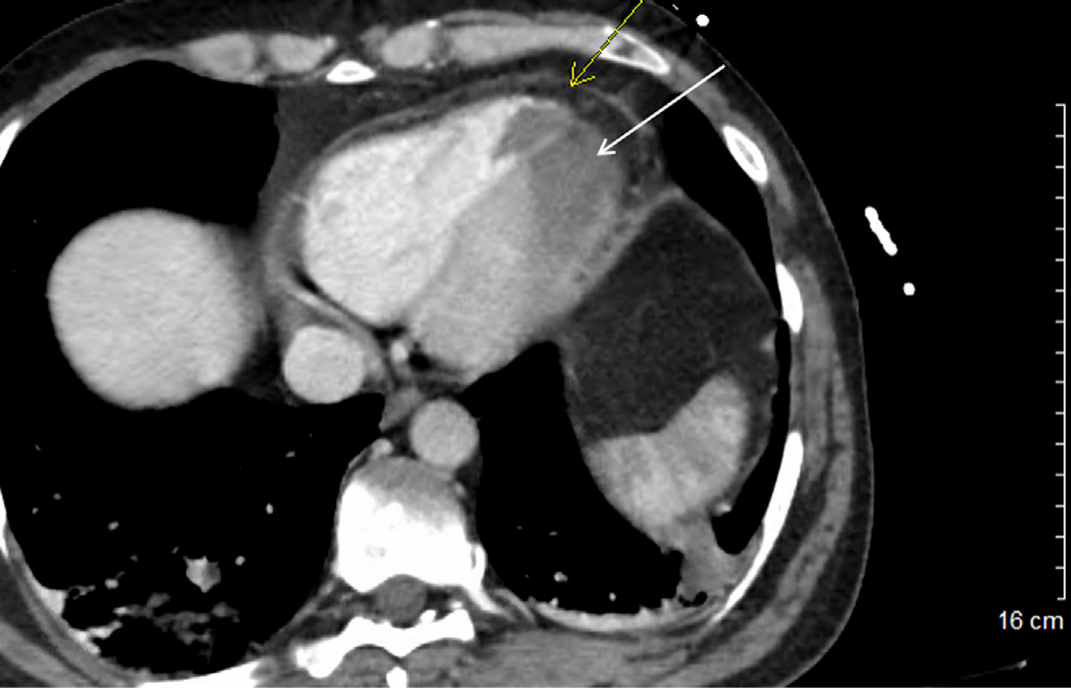
Biventricular Apical Thromboses Computed tomography scan showing left (white arrow) and right (yellow arrow) ventricular apical thromboses, a new finding compared with a computed tomography scan performed 2 months earlier.

Computed tomography angiography showed distal aortic thrombus and right popliteal arterial occlusion. Left and right ventricular apical thromboses were an incidental finding (Figure 2). Immediate coronary and peripheral angiogram revealed acute thrombotic occlusion of proximal-mid left anterior descending artery (LAD) (Video 1) and right popliteal artery (Video 2), respectively. A subsequent echocardiogram confirmed an apical left ventricular thrombus with severely reduced systolic function (Video 3).

## Management

Supplemental oxygenation with 100% fraction of inspired oxygen via mechanical ventilation was initiated. The patient was deemed unstable for hyperbaric oxygenation. Vascular surgery and interventional cardiology staff collaboratively scrubbed for an emergent peripheral and heart catheterization. Due to heavy clot burden, the cardiology team was unable to establish flow in the LAD despite multiple balloon inflations. The vascular surgeon used the same left common femoral access to navigate across the aorta into the right femoral territory using a 7-F sheath. The thrombotic segment was crossed with a Glidewire (Terumo Medical), and a TrailBlazer catheter (Medtronic) was advanced. Angiogram via the TrailBlazer catheter confirmed heavy clot burden in the posterior tibial artery. Although suction thrombectomy with a Lightning 7 catheter (Penumbra Inc) was able to remove some clots, it failed to establish distal flow. A 6 mg bolus of tissue plasminogen activator was administered intravascularly followed by 12 hours of catheter-directed tissue plasminogen activator infusion (EKOS). Twelve hours later, suction thrombectomy was attempted again, but it failed.

**Figure 3:**
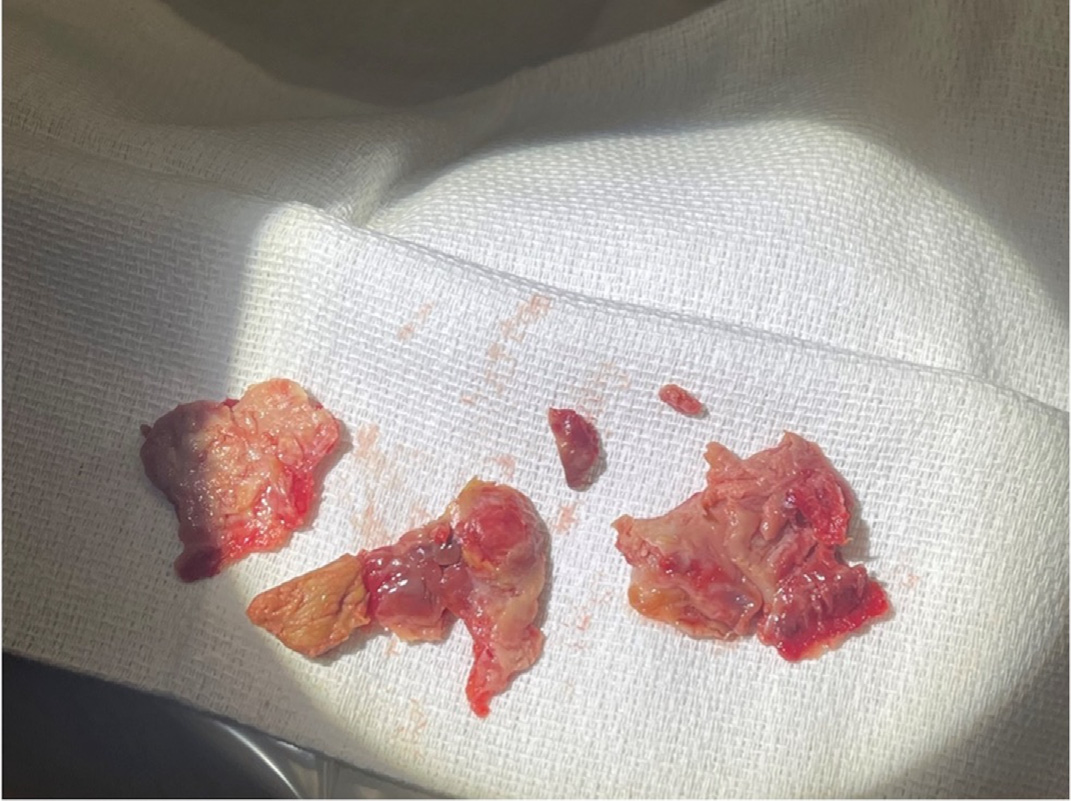
LV Apical Thrombus Removed During LV Apical Aneurysm Repair LV = left ventricular.

Due to concerns regarding postischemic compartment syndrome, the patient underwent urgent above-knee amputation of his right leg. After a prolonged course of multidisciplinary critical care including mechanical ventilation, hemodialysis, and supportive care, the patient was extubated in 1 week. Subsequently, he underwent coronary artery bypass grafting with left internal mammary artery to LAD along with left ventricular apical aneurysm repair and apical thrombus debridement (Figure 3). Although surgical debridement of ventricular thrombus is not conventional, the thrombus burden was so extensive that unless surgically removed, it would have caused significant diastolic dysfunction due to loss of ventricular cavitary volume.

## Discussion

Approximately 100,000 emergency visits related to CO poisoning are reported annually in the United States, highlighting its public health impact.^1^ Although the link between CO poisoning and venous thromboembolism is well documented, cases involving extensive multisystem venous *and* arterial thromboses are rarely reported.^2^

CO is a colorless, odorless gas that binds tightly to hemoglobin, impairing the blood’s oxygen-carrying capacity. CO’s stronger affinity to hemoglobin molecule (relative to oxygen) leads to cellular hypoxia, which in turn can promote thrombosis.^3^ CO exposure can lead to a transient hypercoagulable state causing both venous *and* arterial thromboses as described in this case report.^4^^,^^5^ Moreover, exogenous CO seems to seep in an extravascular manner and cause direct cardiotoxicity.^6^

According to the Centers for Disease Control and Prevention, CO hemoglobin levels >5% in a nonsmoker and >12% in a patient with a history of smoking constitute confirmatory evidence of CO poisoning.^7^ Widespread cases of CO poisoning were reported during hurricane Ike (2008), Hurricane Sandy (2012), and an ice storm in Kentucky (2009). Frequent environmental disasters, financial constraints, and extreme winter temperatures have compelled home dwellers to resort to alternate sources of power, including gasoline generators, camp stoves, and gas-powered tools; these are potential sources of accidental CO poisoning. As noted in this case report, malfunctioning water heaters are a known source of accidental household CO exposure.^8^ Last but not the least, general measures such as installing CO detectors, regular inspection of air/water heating systems, and avoiding indoor usage of portable generators can be lifesaving.

## Follow-Up

The patient is being treated with long-term systemic anticoagulation and hemodialysis. He is alive at 11 months’ follow-up.

## Conclusions

CO poisoning can manifest with myriads of clinical signs and symptoms, most commonly confusion, but cardiovascular consequences should not be overlooked. Clinical vigilance for CO poisoning should be maintained, especially during winter months and environmental calamities. Thrombotic complications from CO poisoning are not a common phenomenon. However, in the context of an underlying hypercoagulable state (in this case, noncompliance with apixaban), CO poisoning may lead to devastating systemic thromboembolism.

## Funding Support and Author Disclosures

This research was supported by the University of Texas Health, San Antonio, Department of Medicine. The authors have reported that they have no relationships relevant to the contents of this paper to disclose.

## Appendix

Video 1 Coronary angiogram showing extensive proximal left anterior descending artery thrombus and mid left anterior descending artery occlusion

Video 2 Peripheral angiogram shows occlusion of popliteal artery

Video 3 Echocardiogram confirms severely reduced left ventricular systolic function, dyskinetic apex, and apical left ventricular thrombus
